# Rare Combination of Pneumothorax, Pneumomediastinum and Pneumopericardium and a Bronchopleural Fistula in Covid-19

**DOI:** 10.1590/0037-8682-0188-2023

**Published:** 2023-07-24

**Authors:** Ewe Jin Koh, Ming Lee Chin

**Affiliations:** 1 Hospital Taiping, Department of Internal Medicine, Malaysia. Hospital Taiping Department of Internal Medicine Malaysia; 2 Hospital Taiping, Department of Medicine-Paediatrics, Malaysia. Hospital Taiping Department of Medicine-Paediatrics Malaysia

A 78-year-old man presented with a one-week history of worsening shortness of breath and cough. On examination, he had obvious respiratory distress and was profoundly hypoxic, requiring high-flow oxygen. Chest radiography revealed severe consolidation in both lung fields (Figure 1)**.**

**FIGURE 1:**
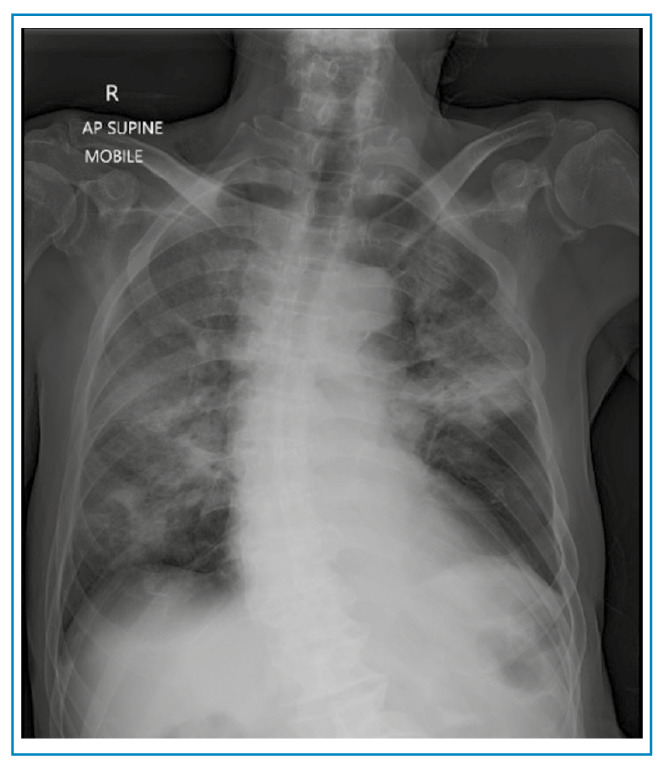
Chest radiograph on admission showing bilateral diffuse ground-glass opacities.

Covid-19 was confirmed by SARS-CoV-2 detection in nasopharyngeal and oropharyngeal swab samples using RT-PCR. Intravenous corticosteroids, immune modulators, and therapeutic anticoagulants were initiated. However, the patient required ongoing high-flow oxygen to maintain oxygen saturation.

Computed tomography (CT) of the thorax revealed ground-glass opacities in both lung fields with a right pneumothorax, pneumopericardium, and pneumomediastinum, and a bronchopleural fistula (Figures 2 and Figure 3). The patient was treated conservatively owing to his frailty. He was discharged from hospital to palliative care. 

**FIGURE 2:**
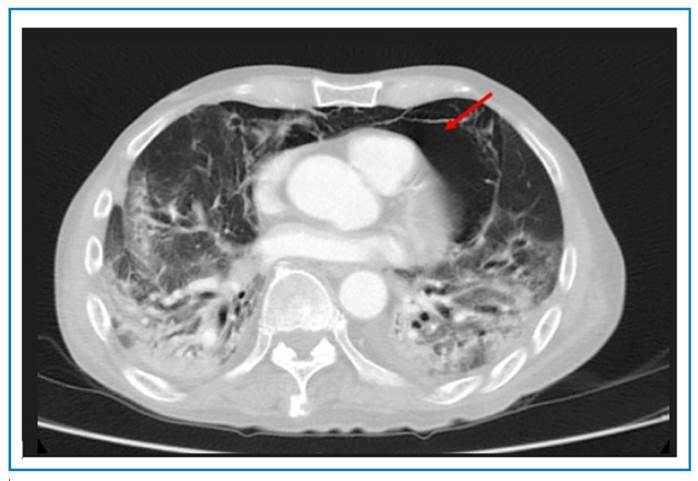
Computed tomography (CT) of the thorax with pneumomediastinum and pneumopericardium shown by the red arrow.

**FIGURE 3:**
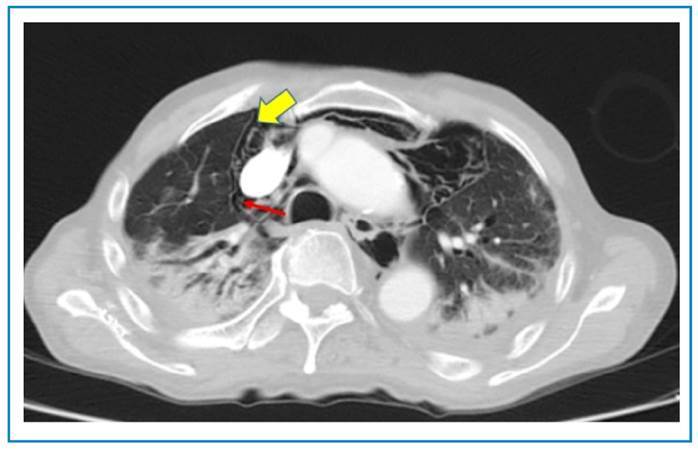
Computed tomography (CT) of the thorax with a small right apical pneumothorax, shown by a yellow arrow. The red arrow shows a bronchopleural fistula.

Bronchopleural fistulas, which cause spontaneous pneumothorax, pneumomediastinum, and pneumopericardium, are rare complications of Covid-19. They can occur because of the overwhelming cytokine storm leading to alveolar rupture and air dissects through the peribronchial vascular sheath into the mediastinum and pericardium^1^.

The patient developed a spontaneous pneumothorax, pneumomediastinum, and pneumopericardium, despite not receiving positive pressure ventilation. To our knowledge this triple combination has not been reported previously in Covid-19. It is important to recognize that they may occur in the absence of positive pressure ventilation. 

Currently, there are no official guidelines for managing these complications in the context of Covid-19. Several patients have been treated conservatively with good outcomes^2^^;^ however some patients require surgical intervention^3^. Further reports are required to obtain further evidence on the management of these complications.

## References

[1] 1. Menter T, Haslbauer JD, Nienhold R, Savic S, Hopfer H, Deigendesch N, et al. Postmortem examination of COVID-19 patients reveals diffuse alveolar damage with severe capillary congestion and variegated findings in lungs and other organs suggesting vascular dysfunction. Histopathology. 2020;77(2):198-209. Available from: http://dx.doi.org/10.1111/his.14134 10.1111/his.14134 PMC7496150 32364264

[2] 2. Chang CY. Pneumomediastinum in a patient with severe Covid-19 pneumonia. Rev Soc Bras Med Trop. 2021;54:e03962021. Available from: http://dx.doi.org/10.1590/0037-8682-0396-2021 10.1590/0037-8682-0396-2021 PMC8313098 34320135

[3] 3. Talon A, Arif MZ, Mohamed H, Khokar A, Saeed AI. Bronchopleural fistula as a complication in a COVID-19 patient managed with endobronchial valves. J Investig Med High Impact Case Rep. 2021;9:23247096211013215. Available from: http://dx.doi.org/10.1177/23247096211013215 10.1177/23247096211013215 PMC8114316 33928804

